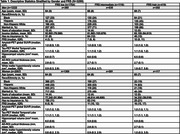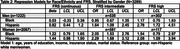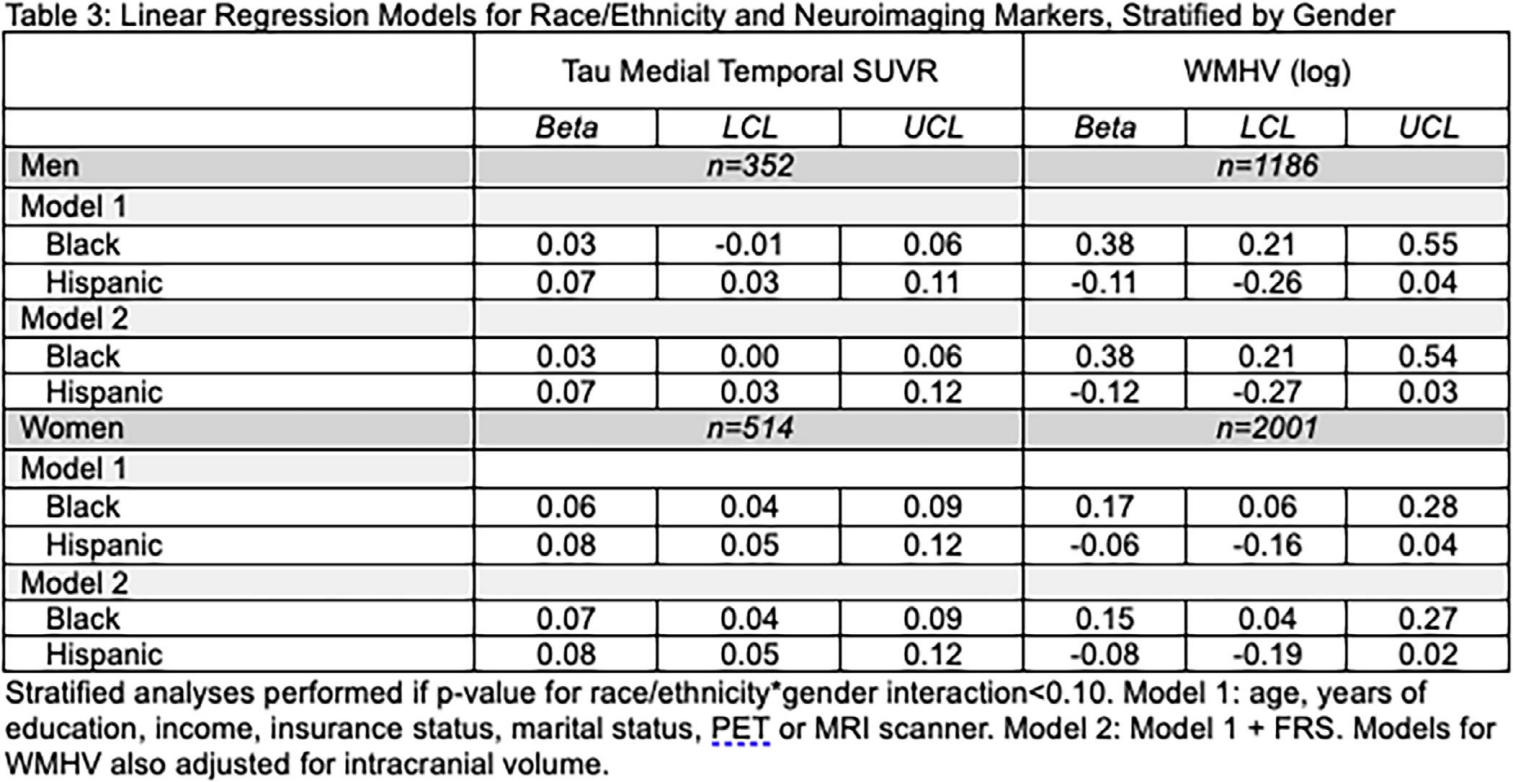# Does Cardiovascular Risk Mediate Associations Between Race/Ethnicity, Gender, and Neuroimaging Markers of Dementia? An Analysis from the HABS‐HD Cohort

**DOI:** 10.1002/alz70860_100000

**Published:** 2025-12-23

**Authors:** Michelle R. Caunca, Amber L Bahorik, Xiaqing Jiang, Meredith N. Braskie, Sid E. O'Bryant, Kristine Yaffe

**Affiliations:** ^1^ Department of Neurology, University of California, San Francisco, San Francisco, CA, USA; ^2^ University of California, San Francisco, San Francisco, CA, USA; ^3^ Imaging Genetics Center, Mark and Mary Stevens Neuroimaging and Informatics Institute, Keck School of Medicine, University of Southern California, Marina del Rey, CA, USA; ^4^ Institute for Translational Research, University of North Texas Health Science Center, Fort Worth, TX, USA; ^5^ Department of Psychiatry, University of California San Francisco, San Francisco, CA, USA; ^6^ University of California, San Francisco and San Francisco VA Health Care System, San Francisco, CA, USA

## Abstract

**Background:**

Emerging data illustrates important differences in dementia‐related pathology by race/ethnicity and gender, similar to patterns in cardiovascular risk. We hypothesized that neuroimaging markers of dementia would differ by race/ethnicity and gender and that cardiovascular risk would partially mediate these associations.

**Methods:**

Using the Health and Brain Study‐Health Disparities data (HABS‐HD), we estimated the impact of racial/ethnic and gender differences on neuroimaging markers of cerebrovascular disease (white matter hyperintensity volume [WMHV]) and neurodegeneration (AD ROI cortical thickness [in mm], global amyloid and medial temporal tau deposition [SUVR]) in dementia‐free participants. Linear and multinomial regression was used, adjusting for age, race/ethnicity, gender, race/ethnicity*gender, years of education, income, insurance status, marital status, and intracranial volume (for WMHV). Stratified analyses were performed if race/ethnicity*gender term *p* <0.10. Framingham Risk Score (FRS) was added to examine mediation by cardiovascular risk.

**Results:**

Among 3289 participants with either MRI or PET data available at their baseline visit (63% women, 26% Black, 37% Hispanic, and 36% non‐Hispanic white), 34% and 13% had an intermediate and high FRS, respectively. Women of color had the highest odds of an intermediate FRS (OR [95% CI], Black: 2.05 [1.53, 2.76], Hispanic: 2.29 [1.68, 3.12]), and Hispanic men had the highest odds of a high FRS (2.78 [1.72, 4.51]), compared to non‐Hispanic whites. Black participants had lower amyloid‐beta SUVR compared to non‐Hispanic whites (ß [95% CI]: ‐0.04 [‐0.07, ‐0.02]). AD ROI cortical thickness did not differ by race/ethnicity and gender. Women of color had greater medial temporal tau SUVR (ß [95% CI], Black: 0.06 [0.04, 0.09], Hispanic: 0.08 [0.05, 0.12]). Associations with amyloid or tau SUVR were stable with adjustment of FRS. Black participants had greater WMHV (ß [95% CI], men: 0.38 [0.21, 0.55], women: 0.17 [0.06, 0.28]), while Hispanic participants had lower WMHV (though not significantly). Adjustment for FRS partially mediated associations for Black women (ß [95% CI], 0.15 [0.04, 0.27]).

**Conclusions:**

Cardiovascular risk and neuroimaging markers of dementia vary jointly by race/ethnicity. Women of color may have a greater likelihood of tau deposition and benefit more from reduction of cardiovascular risk to reduce small vessel disease burden.